# miRNome Expression Analysis Reveals New Players on Leprosy Immune Physiopathology

**DOI:** 10.3389/fimmu.2018.00463

**Published:** 2018-03-09

**Authors:** Claudio Guedes Salgado, Pablo Pinto, Raquel Carvalho Bouth, Angélica Rita Gobbo, Ana Caroline Cunha Messias, Tatiana Vinasco Sandoval, André Mauricio Ribeiro dos Santos, Fabiano Cordeiro Moreira, Amanda Ferreira Vidal, Luiz Ricardo Goulart, Josafá Gonçalves Barreto, Moisés Batista da Silva, Marco Andrey Cipriani Frade, John Stewart Spencer, Sidney Santos, Ândrea Ribeiro-dos-Santos

**Affiliations:** ^1^Laboratório de Dermato-Imunologia, Instituto de Ciências Biológicas (ICB), Universidade Federal do Pará (UFPA), Marituba, Brazil; ^2^Laboratório de Genética Humana e Médica, ICB, UFPA, Belém, Brazil; ^3^Núcleo de Pesquisas em Oncologia (NPO), UFPA, Belém, Brazil; ^4^Laboratório de Nanobiotecnologia, Instituto de Genética e Bioquímica, Universidade Federal de Uberlândia (UFU), Uberlândia, Brazil; ^5^Laboratório de Epidemiologia Espacial (LabEE), Campus Castanhal, UFPA, Belém, Brazil; ^6^Divisão de Dermatologia, Departamento de Clínica Médica da Faculdade de Medicina de Ribeirão Preto, USP, Ribeirão Preto, Brazil; ^7^Mycobacteria Research Laboratories, Department of Microbiology, Immunology and Pathology, Colorado State University, Fort Collins, CO, United States

**Keywords:** leprosy, immunology, Schwann cells, apoptosis, neuropathic pain, microRNA, miRNome, epigenetics

## Abstract

Leprosy remains as a public health problem and its physiopathology is still not fully understood. MicroRNAs (miRNA) are small RNA non-coding that can interfere with mRNA to regulate gene expression. A few studies using DNA chip microarrays have explored the expression of miRNA in leprosy patients using a predetermined set of genes as targets, providing interesting findings regarding the regulation of immune genes. However, using a predetermined set of genes restricted the possibility of finding new miRNAs that might be involved in different mechanisms of disease. Thus, we examined the miRNome of tuberculoid (TT) and lepromatous (LL) patients using both blood and lesional biopsies from classical leprosy patients (LP) who visited the Dr. Marcello Candia Reference Unit in Sanitary Dermatology in the State of Pará and compared them with healthy subjects. Using a set of tools to correlate significantly differentially expressed miRNAs with their gene targets, we identified possible interactions and networks of miRNAs that might be involved in leprosy immunophysiopathology. Using this approach, we showed that the leprosy miRNA profile in blood is distinct from that in lesional skin as well as that four main groups of genes are the targets of leprosy miRNA: (1) recognition and phagocytosis, with activation of immune effector cells, where the immunosuppressant profile of LL and immunoresponsive profile of TT are clearly affected by miRNA expression; (2) apoptosis, with supportive data for an antiapoptotic leprosy profile based on *BCL2, MCL1*, and *CASP8* expression; (3) Schwann cells (SCs), demyelination and epithelial–mesenchymal transition (EMT), supporting a role for different developmental or differentiation gene families, such as Sox, Zeb, and Hox; and (4) loss of sensation and neuropathic pain, revealing that *RHOA, ROCK1, SIGMAR1*, and aquaporin-1 (*AQP1*) may be involved in the loss of sensation or leprosy pain, indicating possible new therapeutic targets. Additionally, *AQP1* may also be involved in skin dryness and loss of elasticity, which are well known signs of leprosy but with unrecognized physiopathology. In sum, miRNA expression reveals new aspects of leprosy immunophysiopathology, especially on the regulation of the immune system, apoptosis, SC demyelination, EMT, and neuropathic pain.

## Introduction

Leprosy is an ancient disease caused by *Mycobacterium leprae*, an obligate intracellular pathogen that infects macrophages and Schwann cells (SCs), resulting in nerve and skin lesions with loss of sensation, the hallmark of the disease (1).

After contact with the bacilli, most people control *M. leprae* multiplication and will never develop leprosy (2). If the bacilli survive, the host may develop two stable polar forms of disease, the paucibacillary (PB) tuberculoid (TT) form or the multibacillary (MB) lepromatous (LL) form, besides the three borderline intermediate unstable forms, borderline TT, borderline borderline, and borderline LL. PB patients have a good cellular immune response (CIR) that may restrict bacillus proliferation, resulting in a few lesions that are usually limited to a specific part of the tegument and to a few nerve trunks. MB patients, on the other hand, have a poor CIR with an exacerbated humoral immune response that is not effective for controlling bacillus proliferation. Patients have many lesions disseminated through the body, including the skin and peripheral nerves (3).

The natural history of the disease results in disabilities. Demyelination caused by SC degeneration is one of the main events in leprosy physiopathology, together with exacerbations of the immune response, known as leprosy reactions. While patients evolve a loss of sensation on the skin, they may also have peripheral nerve neuropathic pain that can be exacerbated by the reactions and may last for many years, even after multidrug therapy (MDT) (4).

Genetic studies of portions of the genome that do not encode protein revealed one class of small non-coding RNAs [named microRNAs (miRNAs)] that are involved in posttranscriptional control of gene expression (5). Knowledge about the interaction between miRNA and leprosy is limited (6–12). A recent study demonstrated that a miRNA can influence the mechanism whereby the cell host can prevent bacillus growth and generate natural barriers against infection by *M. leprae* (9). Evidence has shown that miRNAs are able to modulate host antibacterial pathways during the infection process and influence the outcome of disease (9). Analysis of miRNAs that are differentially expressed in distinct poles of the disease could provide a better understanding of targets for an efficient immune response to prevent infection, as well as elucidate novel possible biomarkers for leprosy, for example, subclinical infection and one possible predictor of who will develop leprosy (13, 14).

Upon contact with *M. leprae*, the human immune system must recognize and process the bacteria to activate immune effector cells. During the interaction, the host cells may be induced to undergo apoptosis, hindering bacillus adaptation or maintaining its survival with attenuated microbicide capacity, to shelter the bacilli (15). Demyelination is a key event in leprosy, and SC are critical for myelin production and maintenance on peripheral nerves. Epithelial–mesenchymal transition (EMT) is a biological process in which specialized cells may undergo a phenotypic change to mesenchymal cells, with higher motility, greater resistance to apoptosis, induction of fibrosis, loss of markers for specialized cells, and the acquisition of new proliferation markers (16). Mechanisms responsible for the loss of sensation and neuropathic pain are poorly understood.

Our work presents the first leprosy miRNome from lesions and blood of LP. In addition to describing the miRNAs, we chose those with significant differential expression, searched for their target genes, and constructed possible pathways based on current knowledge of leprosy immune pathophysiology.

## Materials and Methods

### Study Design and Participants

A total of 28 biological samples from leprosy patients (LP) before starting MDT treatment who attended the Dr. Marcello Candia Reference Unit in Sanitary Dermatology of the State of Pará (UREMC) on 2014, in Marituba, Pará, Brazil, and individuals without leprosy and with no other diseases [healthy subjects (HS)] were included in the present study, according to the following groups: (a) 17 tissue biopsies samples [11 from LP (6 LL and 5 TT) and 6 skin tissue from HS for controls] and (b) 11 peripheral blood samples [9 from LP (5 LL and 4 TT) and 2 from HS for controls]. Table 1 describes gender, age, bacterial index, anti-PGL-I optical density, and disability grading of the 11 LP selected for the study.

**Table 1:** Leprosy patients enrolled on the study: ID, gender, age, bacterial index, anti-PGL-I IgM, and disability grade.

ID^a^	Gender	Age	Bacterial index	Anti-PGL-I^b^	Disability grade
LL 1	M	30	3.25	2.023	0
LL 2	F	81	3.50	1.551	2
LL 3	M	72	5.75	2.145	1
LL 4	M	64	4.25	1.849	2
LL 5	M	51	5.00	1.158	1
LL 6	F	58	4.75	0.792	0
TT 1	M	40	0	0.041	0
TT 2	F	44	0	1.200	0
TT 3	M	37	0	NR	0
TT 4	M	20	0	0.184	0
TT 5	M	19	NR	0.022	0

*^a^The ID is composed of the clinical form followed by a sequential number*.

*^b^Optical density of ELISA*.

*NR, not realized*.

This study adhered to the Declaration of Helsinki and was approved by the Institute of Health Sciences Research Ethics Committee at Universidade Federal do Pará, certified by CAAE 26765414.0.0000.0018. A written informed consent to publish was obtained from every individual who accepted to participate in this study. The small RNAseq number register is ERP105473 on European Nucleotide Archive database.

### Total RNA Storage, Extraction, and Quantification

**Figure 1:**
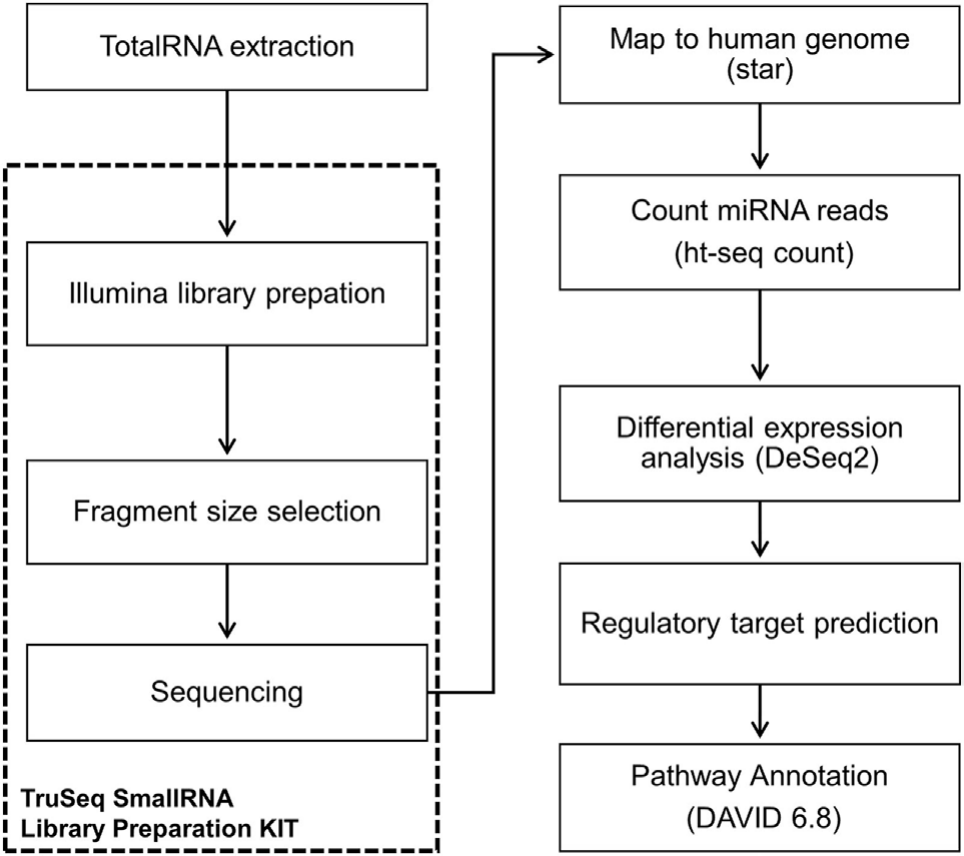
Flowchart showing workflow of miRNome sequencing and analysis.

A flowchart (Figure 1) presents all the steps performed during miRNA Seq (extraction, library, sequencing data processing and analysis pipeline, target gene identification). The whole peripheral blood samples were collected into a Tempus Blood RNA Tube (Thermo Fisher Scientific, USA) and stored at −20°C until extraction. The skin tissue biopsy samples were collected in a propylene tube with 2 mL RNAlater (Thermo Fisher Scientific, USA) and stored in liquid nitrogen until use. Total RNA was extracted from the tissue sample using TRIzol reagent (Invitrogen, USA), and samples were eluted in DEPC water and stored in liquid nitrogen. Total RNA was extracted from blood samples using the MagMAX RNA Isolation Kit (Thermo Fisher Scientific, USA). Total RNA quantity and quality assessed were performed with a NanoDrop 1000 (Thermo Scientific, USA) and Agilent 2200 TapeStation (Agilent Technologies, USA).

### Library Preparation and Next-Generation Sequencing (NGS)

The library was prepared using the TruSeq Small RNA Library Preparation Kit (Illumina, Inc., USA) according to the manufacturer’s instructions, and all samples used for the library had an initial concentration of 1 μg/5 μL of total RNA. The library was validated and quantified with an Agilent 2200 TapeStation (Agilent Technologies, USA) platform and by real time PCR with the KAPA Library Quantification Kit (KAPA BIOSYSTEM, USA). The libraries were then diluted to a concentration of 4 nM and sequenced using the MiSeq Reagent Kit v3 150 cycle (Illumina, Inc., USA) on a MiSeq System (Illumina, Inc., USA). The tissue and blood samples were sequenced separately.

### Sequencing Data Processing and Analysis-Small RNA-Seq Pipeline

The sequencing data were processed on an Illumina MiSeq reporter and extracted in FASTQ format. A pipeline of pre-processing using the Fastx_toolkit was applied for a low filter quality, trimers of extreme 3′ reads and contaminant removal. The pipeline was performed according the chronogram: (a) average Phred quality score (*Q*) greater than 30, (b) reads more than 17 nucleotides long, and (c) base calling error probabilities (*P*) greater than 80. Next, read alignment with the human genome (GRCh37) in combination with the miRNA data base (MirBase v.19) was performed using Spliced Transcripts Alignment to a Reference. The miRNA was scored with htseq-count toll, and the results were normalized and analyzed using the Bioconductor-*DESeq2* package with R statistical software. Thus, the following comparisons were conducted: (a) LP vs. HS; (b) TT leprosy vs. HS; (c) LL leprosy vs. HS; (d) TT vs. LL leprosy. Adjusted values of *p* ≤ 0.05 and a log2 fold change >2 were considered statistically significant.

### Target Gene Identification

The genes regulated by the differentially expressed miRNAs detected during the analysis were identified using four tools:(i) TargetCompare (http://lghm.ufpa.br/ferramentas-de-estudos/targetcompare/) (17); (ii) miRTarBase (http://mirtarbase.mbc.nctu.edu.tw); (iii) DIANA miRPath v.3 (18); and (iv) TargetScan (19). We selected only genes regulated by two or more miRNAs with strong experimental evidences support, such as those confirmed by western blotting, reporter assay or qPCR.

Selected targets were further investigated using the pathway enrichment tool DAVID 6.8 (20) that provides a comprehensive set of functional annotation tools and searches in BioCarta and KEGG pathway maps to help investigators to understand biological meaning behind a large list of genes. The pathway enrichment analysis was performed separately for the following groups: (i) HS vs. LP downregulated miRNAs in tissue; (ii) HS vs. LL downregulated miRNAs in tissue; (iii) HS vs. LL upregulated miRNAs in tissue; and (iv) HS vs. LP downregulated miRNAs in blood.

## Results

This study evaluated two types of leprosy samples, skin biopsies and blood by distinct NGS. For the two sample types, the differential expression profiles of the miRNAs were analyzed to identify possible leprosy biomarkers to assist our understanding of epigenetic control mechanisms of the immune response, apoptosis, SC demyelination, EMT, and neuropathic pain.

### miRNA Sequencing and Differential Expression Profiles of Tissue Samples

This sequencing yielded 4 million reads. After the process pipeline, more than 96% of the reads were aligned with the human genome, and the miRNA count was performed using *htseq-count* (miRNA count ≥ 10), with an average of 36,745 reads per sample and 656 miRNAs expressed in at least one sample.

**Figure 2:**
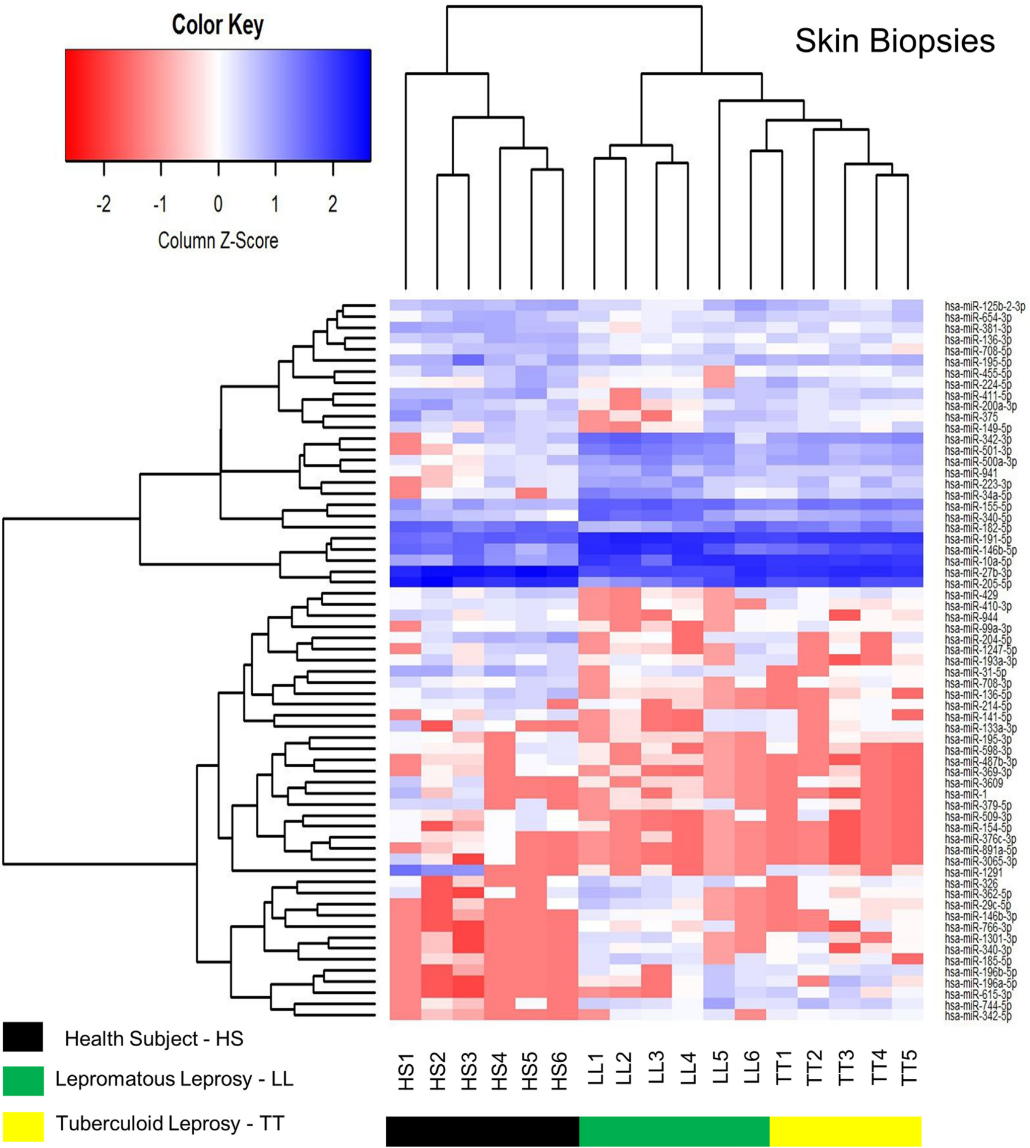
Heatmap of skin biopsies for the differentially expressed microRNAs (miRNAs) among health subjects (HS), lepromatous (LL) leprosy, and tuberculoid (TT) leprosy. It represents the RPKM *z*-score of 67 differentially expressed miRNAs. miRNAs (rows) were hierarchically clustered according their expression in the three different samples (HS, LL, and TT). The clustering was able to distinguish HS from leprosy patients, as well as LL from TT.

A heatmap was constructed using the RPKM (Reads per Kilobases per Million) expression for all differentially expression miRNA (Figure 2). The analysis identified the RPKM z-score of 67 differentially expressed miRNAs, 43 downregulated and 24 upregulated (Table 2; Data Sheet S1 in Supplementary Material) in skin biopsies among HS, LL, and TT. miRNAs (rows) were hierarchically clustered according their expression, and organized according to the three groups, HS, LL, and TT (columns). A hierarchical clustering of the data illustrates how those markers were able to distinguish HS from leprosy patients in general, and LL from TT poles.

**Table 2:** Number of miRNAs that were differentially expressed in LP (TT and LL) compared with HS in skin biopsy samples.

Analysis	miRNAs	miRNAs downregulated	miRNAs upregulated
LP vs. HS	43	26	17
TT vs. HS	14	7	7
LL vs. HS	60	41	19

*LP, leprosy patients; TT, tuberculoid; LL, lepromatous; HS, health subjects*.

**Figure 3:**
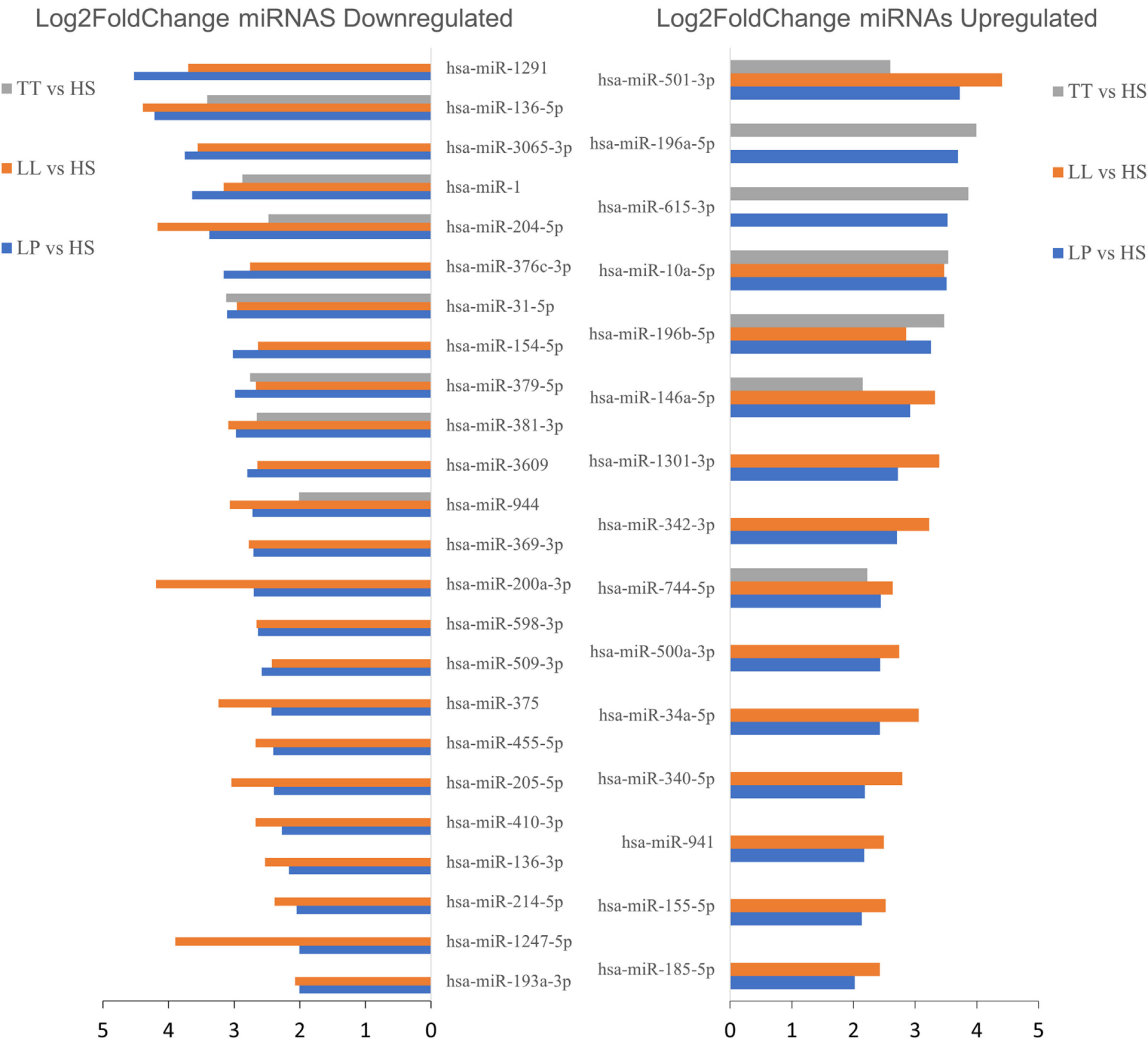
Upregulated or downregulated microRNAs (miRNAs) in at least two comparisons [tuberculoid (TT) vs. healthy subjects (HS), lepromatous (LL) vs. HS, or leprosy patients (LP) vs. HS] on skin biopsies samples. Among the 67 differentially expressed miRNAs (|log2 fold change| > 2 and adjusted *p*-value < 0.05), here we highlight 39 miRNAs differentially expressed between HS versus LP and HS versus LL or TT patients, each indicated by blue, orange, and gray bars, respectively. The barplot represents each miRNA (*y*-axis) absolute log2 fold change (*x*-axis) for each comparison and separated in up- and downregulated miRNA regarding HS.

Figure 3 shows the 39 simultaneous differentially expressed miRNAs, for at least two of the three comparisons conducted as described in Table 2 (HS vs. LP, HS vs. TT, and HS vs. LL), organized by their fold change showing 24 downregulated and 15 upregulated miRNAs.

**Figure 4:**
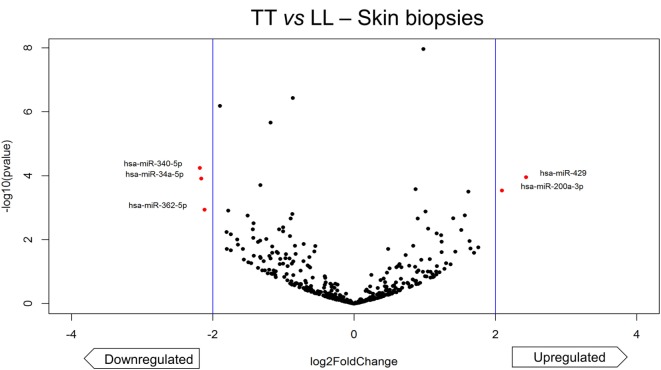
Volcano plot of differentially expressed microRNAs (miRNAs) of skin biopsies between tuberculoid (TT) leprosy and lepromatous (LL) leprosy. The plot represents each miRNA differential expression analysis result where the y-axis is −log_10_ *p*-value of the comparison and the *x*-axis is log_2_ fold-change (*x*-axis) regarding LL. Differentially expressed miRNAs were considered only if the analysis showed |log2 fold-change| > 2 (indicated by the blue vertical lines) and adjusted *p*-value < 0.05. We found five differentially expressed miRNA (highlighted in red), two upregulated (*hsa-miR-429, hsa-miR-200a-3p*) and three downregulated (*hsa-miR-340-5p, hsa-miR-34a-5p, hsa-miR-362-5p*).

The comparison of extreme poles of leprosy, TT and LL, revealed five differentially expressed miRNAs, of which three were downregulated (*hsa-miR-340-5p, hsa-miR-34a-5p, hsa-miR-362-5p*) and two were upregulated (*hsa-miR-429, hsa-miR-200a-3p*). The *hsa-miR-362-5p* appeared only when TT and LL were compared, but not when LP were compared to HS. The volcano plot shows the only five miRNAs differentially expressed (Figure 4).

### miRNA Sequencing and Differential Expression Profiles of Whole Blood Samples

Sequencing yielded 6 million reads. After the process pipeline, more than 95% of the reads were aligned with the human genome, and the miRNAs were counted using *htseq-count* (miRNA count ≥ 10), with an average of 371,325 reads per sample and 527 miRNAs expressed in at least one sample.

**Figure 5:**
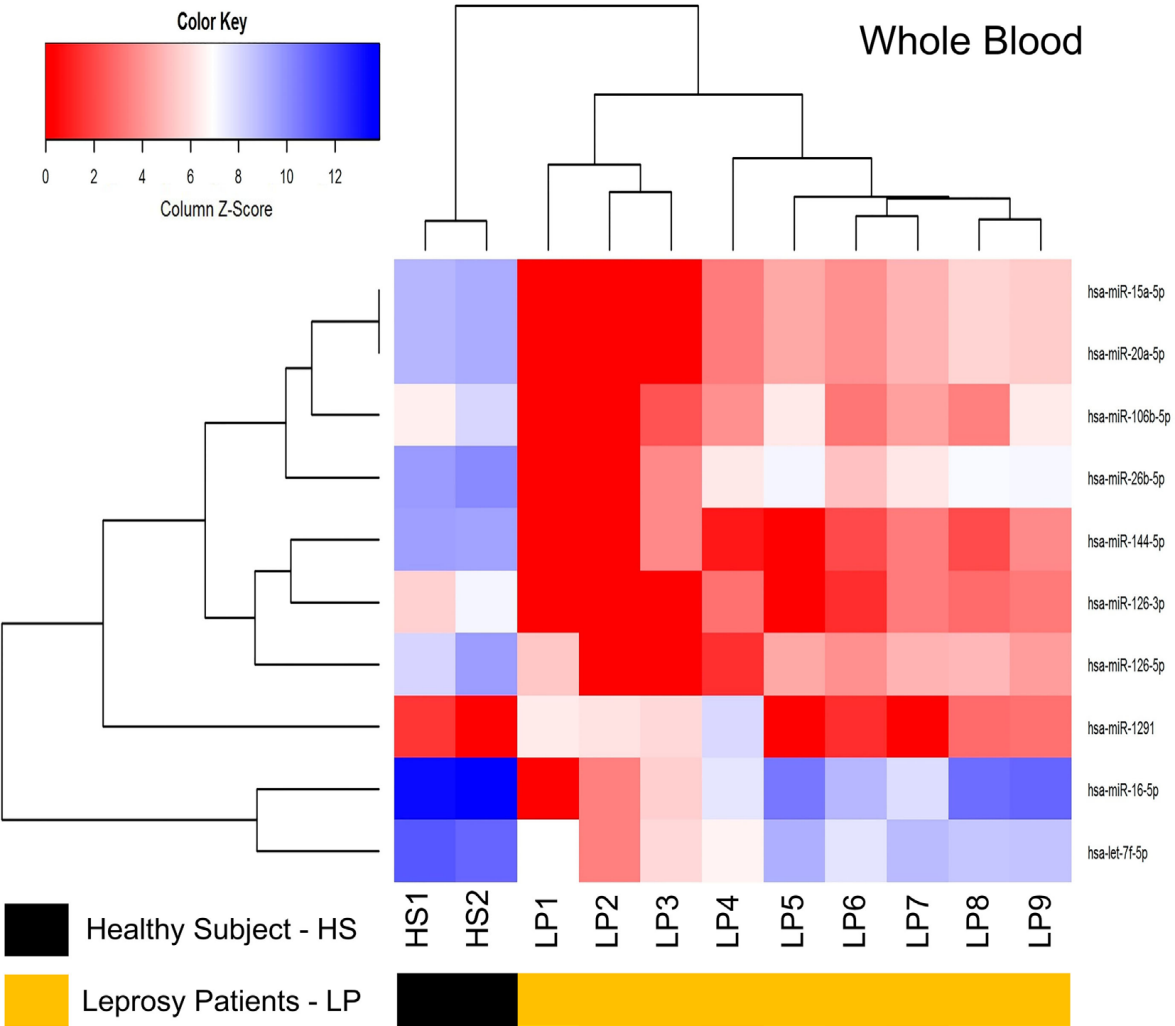
Heatmap of whole blood for the differentially expressed microRNAs (miRNAs) between healthy subjects (HS) and leprosy patients (LP). It represents the RPKM *z*-score of 10 differentially expressed miRNAs. miRNAs (rows) were hierarchically clustered according their expression on HS or LP (columns). The clustering was able to distinguish LP from HS.

The differential expression analysis of blood miRNAs was conducted similarly to that applied for the tissue and revealed a total of 10 differentially expressed miRNAs, with nine downregulated (*hsa-let-7f-5p, hsa-miR-126-3p, hsa-miR-126-5p, hsa-miR-144-5p, hsa-miR-15a-5p, hsa-miR-20a-5p, hsa-miR-26b-5p, hsa-miR-106b-5p, hsa-miR-16-5p*) and one upregulated (*hsa-miR-1291*) (Table 3; Data Sheet S2 in Supplementary Material). From the differentially expressed miRNAs identified, a heatmap was constructed using RPKM expression and two clusters were observed, with standards of expression able to differentiate LP of HS (Figure 5), although comparisons between TT vs. LL showed no differentially expressed miRNAs.

**Table 3:** Number of miRNAs that were differentially expressed in LP (TT and LL) compared with HS in blood.

Analysis	miRNAs	miRNAs downregulated	miRNAs upregulated
LP vs. HS	7	7	0
TT vs. HS	5	5	0
LL vs. HS	4	3	1

*LP, leprosy patients; TT, tuberculoid leprosy; LL, lepromatous leprosy; HS, health subjects*.

### Target Gene Identification

Using the differentially expressed miRNAs from either blood or skin lesions, we investigated the genes regulated by them separately for up and down regulated miRNAs. The miRNAs and their targets are described in Tables 4–8, as follow: (i) HS vs. LP downregulated (Table 4) and upregulated (Table 5) miRNAs in skin biopsies; (ii) HS vs. LL downregulated (Table 6) and upregulated (Table 7) miRNAs in skin biopsies; and (iii) HS vs. LP downregulated miRNAs in blood (Table 8).

**Table 4:** List of the genes targeted by two or more differentially expressed miRNAs among the 26 downregulated miRNAs in LP vs. HS skin biopsies.

Target gene^a^	MicroRNA	Number of miRNAs
*BCL2*	*hsa-miR-136-5p, hsa-miR-204-5p, hsa-miR-375, hsa-miR-205-5p*	4
*ERBB2*	*hsa-miR-375, hsa-miR-205-5p, hsa-miR-193a-3p*	3
*MET*	*hsa-miR-1-3p, hsa-miR-31-5p, hsa-miR-410-3p*	3
*ABCC1*	*hsa-miR-1291, hsa-miR-1-3p*	2
*ARID1A*	*hsa-miR-1-3p, hsa-miR-31-5p*	2
*BDNF*	*hsa-miR-204-5p, hsa-miR-1-3p*	2
*CDC42*	*hsa-miR-204-5p, hsa-miR-375*	2
*DDX5*	*hsa-miR-1-3p, hsa-miR-205-5p*	2
*ETS1*	*hsa-miR-1-3p, hsa-miR-31-5p*	2
*GRB2*	*hsa-miR-200a-3p, hsa-miR-376c-3p*	2
*IGF1R*	*hsa-miR-375, hsa-miR-376c-3p*	2
*IL11*	*hsa-miR-204-5p, hsa-miR-379-5p*	2
*ITGA5*	*hsa-miR-205-5p, hsa-miR-31-5p*	2
*LRP1*	*hsa-miR-1-3p, hsa-miR-205-5p*	2
*MTDH*	*hsa-miR-136-5p, hsa-miR-375*	2
*PIK3CA*	*hsa-miR-375, hsa-miR-1-3p*	2
*PRKCE*	*hsa-miR-1-3p, hsa-miR-31-5p*	2
*PTEN*	*hsa-miR-200a-3p, hsa-miR-205-5p*	2
*RHOA*	*hsa-miR-375, hsa-miR-31-5p*	2
*SIGMAR1*	*hsa-miR-1-3p, hsa-miR-205-5p*	2
*SMAD4*	*hsa-miR-204-5p, hsa-miR-205-5p*	2
*SNAI2*	*hsa-miR-204-5p, hsa-miR-1-3p*	2
*SOX9*	*hsa-miR-1247-5p, hsa-miR-1-3p*	2
*SP1*	*hsa-miR-375, hsa-miR-1-3p*	2
*SRC*	*hsa-miR-205-5p, hsa-miR-31-5p*	2
*SRF*	*hsa-miR-200a-3p, hsa-miR-1-3p*	2
*TGFBR1*	*hsa-miR-204-5p, hsa-miR-376c-3p*	2
*TP53*	*hsa-miR-200a-3p, hsa-miR-375*	2
*WASF3*	*hsa-miR-200a-3p, hsa-miR-31-5p*	2
*YAP1*	*hsa-miR-200a-3p, hsa-miR-375*	2
*YWHAZ*	*hsa-miR-375, hsa-miR-1-3p*	2
*YY1*	*hsa-miR-205-5p, hsa-miR-31-5p*	2
*ZEB1*	*hsa-miR-200a-3p, hsa-miR-205-5p*	2
*ZEB2*	*hsa-miR-200a-3p, hsa-miR-205-5p*	2

*^a^Target gene with strong evidence only*.

*LP, leprosy patients; HS, health subjects*.

**Table 5:** List of the genes that were potentially targeted by two or more differentially expressed miRNAs among the 17 upregulated miRNAs in LP vs. HS skin biopsies.

Target gene^a^	MicroRNA	Number of miRNAs
*MYC*	*hsa-miR-34a-5p, hsa-miR-155-5p, hsa-miR-744-5p*	3
*RHOA*	*hsa-miR-340-5p, hsa-miR-185-5p, hsa-miR-155-5p*	3
*AR*	*hsa-miR-34a-5p, hsa-miR-185-5p*	2
*BACH1*	*hsa-miR-155-5p, hsa-miR-196a-5p*	2
*BMP7*	*hsa-miR-34a-5p, hsa-miR-342-3p*	2
*CCND1*	*hsa-miR-34a-5p, hsa-miR-155-5p*	2
*CDK6*	*hsa-miR-34a-5p, hsa-miR-185-5p*	2
*CEBPB*	*hsa-miR-34a-5p, hsa-miR-155-5p*	2
*CSF1R*	*hsa-miR-34a-5p, hsa-miR-155-5p*	2
*DNMT1*	*hsa-miR-185-5p, hsa-miR-342-3p*	2
*FADD*	*hsa-miR-155-5p, hsa-miR-146a-5p*	2
*FAS*	*hsa-miR-196b-5p, hsa-miR-146a-5p*	2
*HMGA1*	*hsa-miR-185-5p, hsa-miR-196a-5p*	2
*HNF4A*	*hsa-miR-34a-5p, hsa-miR-766-3p*	2
*HOXB7*	*hsa-miR-196b-5p, hsa-miR-196a-5p*	2
*HOXB8*	*hsa-miR-196b-5p, hsa-miR-196a-5p*	2
*HOXC8*	*hsa-miR-196b-5p, hsa-miR-196a-5p*	2
*ICAM1*	*hsa-miR-155-5p, hsa-miR-146a-5p*	2
*IL8*	*hsa-miR-155-5p, hsa-miR-146a-5p*	2
*KRAS*	*hsa-miR-340-5p, hsa-miR-155-5p*	2
*L1CAM*	*hsa-miR-34a-5p, hsa-miR-146a-5p*	2
*MECP2*	*hsa-miR-340-5p, hsa-miR-155-5p*	2
*MEIS1*	*hsa-miR-155-5p, hsa-miR-196b-5p*	2
*MET*	*hsa-miR-340-5p, hsa-miR-34a-5p*	2
*MTA2*	*hsa-miR-34a-5p, hsa-miR-146a-5p*	2
*MYB*	*hsa-miR-34a-5p, hsa-miR-155-5p*	2
*RAC1*	*hsa-miR-155-5p, hsa-miR-146a-5p*	2
*RDX*	*hsa-miR-196b-5p, hsa-miR-196a-5p*	2
*ROCK1*	*hsa-miR-340-5p, hsa-miR-146a-5p*	2
*SMAD2*	*hsa-miR-155-5p, hsa-miR-146a-5p*	2
*SMAD4*	*hsa-miR-155-5p, hsa-miR-146a-5p*	2
*SOX2*	*hsa-miR-340-5p, hsa-miR-34a-5p*	2
*SPI1*	*hsa-miR-34a-5p, hsa-miR-155-5p*	2
*SREBF1*	*hsa-miR-185-5p, hsa-miR-342-3p*	2
*SREBF2*	*hsa-miR-185-5p, hsa-miR-342-3p*	2
*VEGFA*	*hsa-miR-34a-5p, hsa-miR-185-5p*	2

*^a^Target gene with strong evidence only*.

*LP, leprosy patients; HS, health subjects*.

**Table 6:** List of the genes that were potentially targeted by two or more differentially expressed miRNAs among the 34 specific downregulated miRNAs in LL vs. HS skin biopsies.

Target gene^a^	MicroRNA	Number of miRNAs
*BCL2*	*hsa-miR-375, hsa-miR-205-5p, hsa-miR-429, hsa-miR-182-5p, hsa-miR-195-5p, hsa-miR-708-5p, hsa-miR-224-5p*	7
*CDC42*	*hsa-miR-375, hsa-miR-133a-3p, hsa-miR-195-5p, hsa-miR-224-5p*	4
*IGF1R*	*hsa-miR-375, hsa-miR-376c-3p, hsa-miR-133a-3p, hsa-miR-125b-2-3p*	4
*PTEN*	*hsa-miR-200a-3p, hsa-miR-205-5p, hsa-miR-429, hsa-miR-182-5p*	4
*ZEB2*	*hsa-miR-200a-3p, hsa-miR-205-5p, hsa-miR-429, hsa-miR-708-5p*	4
*ERBB2*	*hsa-miR-375, hsa-miR-205-5p, hsa-miR-193a-3p*	3
*EZH2*	*hsa-miR-200a-3p, hsa-miR-429, hsa-miR-708-5p*	3
*MYB*	*hsa-miR-200a-3p, hsa-miR-429, hsa-miR-195-5p*	3
*SMAD4*	*hsa-miR-205-5p, hsa-miR-182-5p, hsa-miR-224-5p*	3
*SP1*	*hsa-miR-375, hsa-miR-133a-3p, hsa-miR-149-5p*	3
*VEGFA*	*hsa-miR-205-5p, hsa-miR-133a-3p, hsa-miR-195-5p*	3
*ZEB1*	*hsa-miR-200a-3p, hsa-miR-205-5p, hsa-miR-429*	3
*AKT1*	*hsa-miR-199a-3p, hsa-miR-708-5p*	2
*BAP1*	*hsa-miR-200a-3p, hsa-miR-429*	2
*BIRC5*	*hsa-miR-195-5p, hsa-miR-708-5p*	2
*CAB39*	*hsa-miR-375, hsa-miR-195-5p*	2
*CCND1*	*hsa-miR-195-5p, hsa-miR-708-5p*	2
*CCND2*	*hsa-miR-154-5p, hsa-miR-182-5p*	2
*CD44*	*hsa-miR-199a-3p, hsa-miR-708-5p*	2
*CDK6*	*hsa-miR-200a-3p, hsa-miR-195-5p*	2
*CDKN1A*	*hsa-miR-182-5p, hsa-miR-654-3p*	2
*DICER1*	*hsa-miR-154-5p, hsa-miR-195-5p*	2
*DNMT1*	*hsa-miR-200a-3p, hsa-miR-429*	2
*ELMO2*	*hsa-miR-200a-3p, hsa-miR-429*	2
*ERBB2IP*	*hsa-miR-200a-3p, hsa-miR-429*	2
*GRB2*	*hsa-miR-200a-3p, hsa-miR-376c-3p*	2
*HOXB5*	*hsa-miR-200a-3p, hsa-miR-429*	2
*KLF11*	*hsa-miR-200a-3p, hsa-miR-429*	2
*KLHL20*	*hsa-miR-200a-3p, hsa-miR-429*	2
*MAPK14*	*hsa-miR-200a-3p, hsa-miR-199a-3p*	2
*MCL1*	*hsa-miR-193a-3p, hsa-miR-133a-3p*	2
*MET*	*hsa-miR-410-3p, hsa-miR-199a-3p*	2
*PARP1*	*hsa-miR-375, hsa-miR-708-5p*	2
*PHLPP1*	*hsa-miR-375, hsa-miR-224-5p*	2
*PTPRD*	*hsa-miR-200a-3p, hsa-miR-429*	2
*RASSF2*	*hsa-miR-200a-3p, hsa-miR-429*	2
*RIN2*	*hsa-miR-200a-3p, hsa-miR-429*	2
*SEPT7*	*hsa-miR-200a-3p, hsa-miR-429*	2
*SHC1*	*hsa-miR-200a-3p, hsa-miR-429*	2
*TCF7L1*	*hsa-miR-200a-3p, hsa-miR-429*	2
*TP53*	*hsa-miR-200a-3p, hsa-miR-375*	2
*VAC14*	*hsa-miR-200a-3p, hsa-miR-429*	2
*WASF3*	*hsa-miR-200a-3p, hsa-miR-429*	2
*WDR37*	*hsa-miR-200a-3p, hsa-miR-429*	2
*YAP1*	*hsa-miR-200a-3p, hsa-miR-375*	2
*ZFPM2*	*hsa-miR-200a-3p, hsa-miR-429*	2

*^a^Target gene with strong evidence only*.

*LL, lepromatous leprosy; HS, health subjects*.

**Table 7:** List of genes potentially targeted by two or more differentially expressed miRNAs among the 14 specific upregulated miRNAs LL vs. HS skin biopsies.

Target gene^a^	MicroRNA	Number of miRNAs
*CCND1*	*hsa-miR-34a-5p, hsa-miR-155-5p*	3
*CDK6*	*hsa-miR-34a-5p, hsa-miR-185-5p, hsa-miR-191-5p*	3
*CEBPB*	*hsa-miR-34a-5p, hsa-miR-155-5p, hsa-miR-191-5p*	3
*RHOA*	*hsa-miR-340-5p, hsa-miR-185-5p, hsa-miR-155-5p*	3
*AR*	*hsa-miR-34a-5p, hsa-miR-185-5p*	2
*BMP7*	*hsa-miR-34a-5p, hsa-miR-342-3p*	2
*CSF1R*	*hsa-miR-34a-5p, hsa-miR-155-5p*	2
*DNMT1*	*hsa-miR-185-5p, hsa-miR-342-3p*	2
*E2F1*	*hsa-miR-34a-5p, hsa-miR-223-3p*	2
*FOXO3*	*hsa-miR-155-5p, hsa-miR-223-3p*	2
*IGF1R*	*hsa-miR-185-5p, hsa-miR-223-3p*	2
*KIT*	*hsa-miR-34a-5p, hsa-miR-146b-5p*	2
*KRAS*	*hsa-miR-340-5p, hsa-miR-155-5p*	2
*MDM4*	*hsa-miR-34a-5p, hsa-miR-191-5p*	2
*MECP2*	*hsa-miR-340-5p, hsa-miR-155-5p*	2
*MET*	*hsa-miR-340-5p, hsa-miR-34a-5p*	2
*MMP16*	*hsa-miR-155-5p, hsa-miR-146b-5p*	2
*MYB*	*hsa-miR-34a-5p, hsa-miR-155-5p*	2
*MYC*	*hsa-miR-34a-5p, hsa-miR-155-5p*	2
*NOTCH1*	*hsa-miR-34a-5p, hsa-miR-326*	2
*NOTCH2*	*hsa-miR-34a-5p, hsa-miR-326*	2
*PDGFRA*	*hsa-miR-34a-5p, hsa-miR-146b-5p*	2
*SCARB1*	*hsa-miR-185-5p, hsa-miR-223-3p*	2
*SOX2*	*hsa-miR-340-5p, hsa-miR-34a-5p*	2
*SPI1*	*hsa-miR-34a-5p, hsa-miR-155-5p*	2
*SREBF1*	*hsa-miR-185-5p, hsa-miR-342-3p*	2
*SREBF2*	*hsa-miR-185-5p, hsa-miR-342-3p*	2
*VEGFA*	*hsa-miR-34a-5p, hsa-miR-185-5p*	2

*^a^Target gene with strong evidence only*.

*LL, lepromatous leprosy; HS, health subjects*.

**Table 8:** List of genes that were potentially targeted by two or more differentially expressed miRNAs among the nine downregulated miRNAs in LP vs. HS blood.

Target gene^a^	MicroRNA	Number of miRNAs
*CCND1*	*hsa-let-7f-5p, hsa-miR-15a-5p, hsa-miR-20a-5p, hsa-miR-106b-5p, hsa-miR-16-5p*	5
*BCL2*	*hsa-miR-126-3p, hsa-miR-15a-5p, hsa-miR-20a-5p, hsa-miR-16-5p*	4
*PURA*	*hsa-miR-15a-5p, hsa-miR-16-5p, hsa-miR-106b-5p, hsa-miR-20a-5p*	4
*APP*	*hsa-miR-15a-5p, hsa-miR-20a-5p, hsa-miR-106b-5p*	3
*CCND2*	*hsa-miR-15a-5p, hsa-miR-20a-5p, hsa-miR-106b-5p*	3
*CCNE1*	*hsa-miR-15a-5p, hsa-miR-26b-5p, hsa-miR-16-5p*	3
*PTEN*	*hsa-miR-20a-5p, hsa-miR-106b-5p, hsa-miR-26b-5p*	3
*RB1*	*hsa-miR-20a-5p, hsa-miR-106b-5p, hsa-miR-26b-5p*	3
*WEE1*	*hsa-miR-20a-5p, hsa-miR-16-5p, hsa-miR-106b-5p*	3
*ADAM9*	*hsa-miR-126-3p, hsa-miR-126-5p*	2
*AKT3*	*hsa-miR-15a-5p, hsa-miR-16-5p*	2
*BMI1*	*hsa-miR-15a-5p, hsa-miR-16-5p*	2
*BRCA1*	*hsa-miR-15a-5p, hsa-miR-16-5p*	2
*CADM1*	*hsa-miR-15a-5p, hsa-miR-16-5p*	2
*CDK6*	*hsa-miR-26b-5p, hsa-miR-16-5p*	2
*CDKN1A*	*hsa-miR-20a-5p, hsa-miR-106b-5p*	2
*CHORDC1*	*hsa-miR-26b-5p, hsa-miR-16-5p*	2
*CHUK*	*hsa-miR-15a-5p, hsa-miR-16-5p*	2
*E2F1*	*hsa-miR-20a-5p, hsa-miR-106b-5p*	2
*E2F3*	*hsa-miR-106b-5p, hsa-miR-20a-5p*	2
*HMGA1*	*hsa-miR-15a-5p, hsa-miR-16-5p*	2
*HMGA2*	*hsa-miR-16-5p, hsa-miR-15a-5p*	2
*IFNG*	*hsa-miR-16-5p, hsa-miR-15a-5p*	2
*IGF1R*	*hsa-miR-16-5p, hsa-miR-26b-5p*	2
*PTGS2*	*hsa-miR-26b-5p, hsa-miR-16-5p*	2
*RBL1*	*hsa-miR-106b-5p, hsa-miR-20a-5p*	2
*RBL2*	*hsa-miR-20a-5p, hsa-miR-106b-5p*	2
*STAT3*	*hsa-miR-20a-5p, hsa-miR-106b-5p*	2
*SMAD7*	*hsa-miR-20a-5p, hsa-miR-106b-5p*	2
*VEGFA*	*hsa-miR-106b-5p, hsa-miR-16-5p*	2

*^a^Target gene with strong evidence only*.

*LP, leprosy patients; HS, health subjects*.

## Discussion

### Recognition, Engulfment, and Activation of Immune Effector Cells

**Figure 6:**
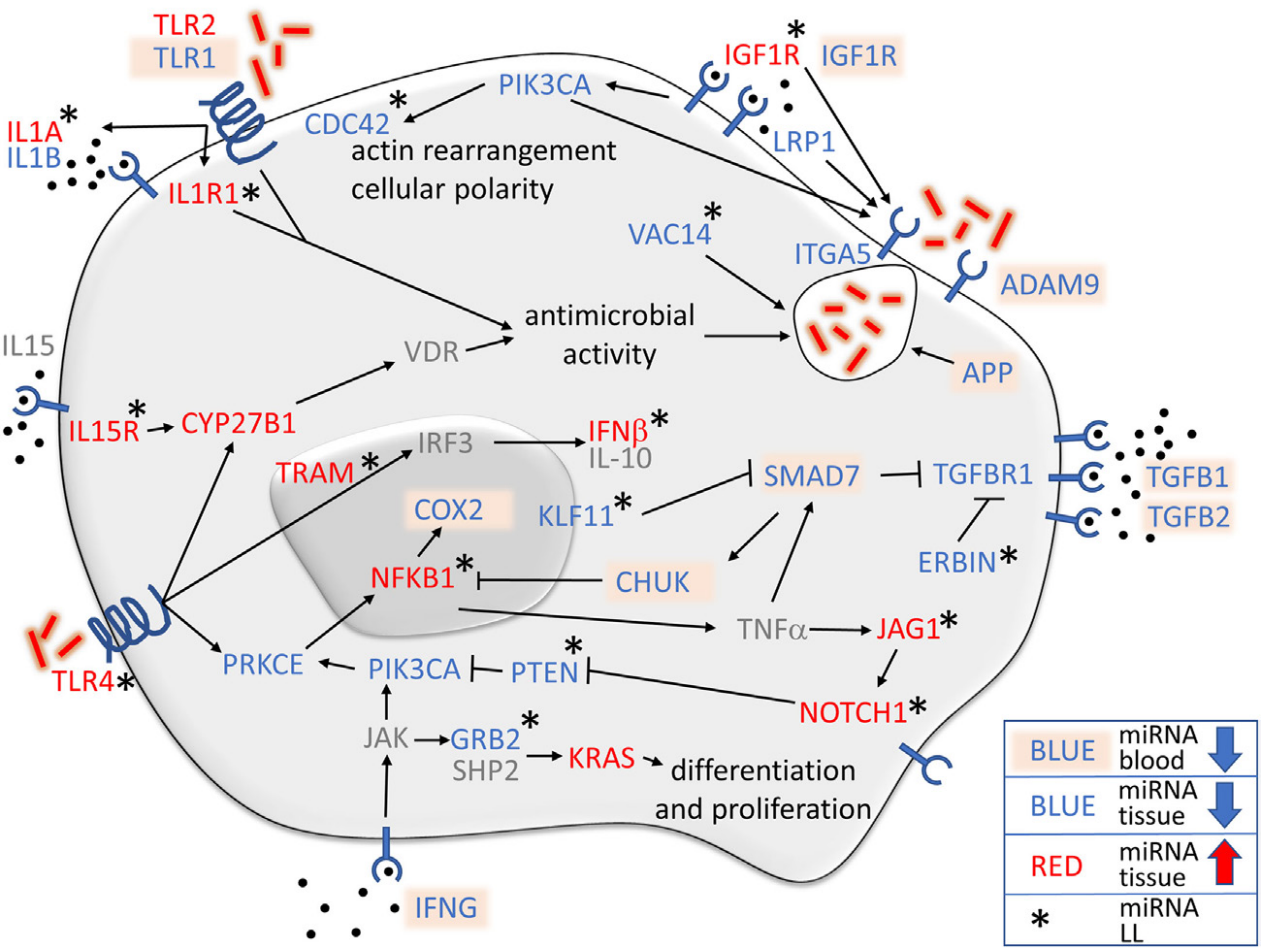
Overview of the relationship of monocyte immunology to microRNAs (miRNAs) regulating genes in leprosy lesions and blood. Most of the genes controlled by the miRNAs were found in lesional tissue, shown either in blue (downregulated miRNAs) or in red (upregulated miRNAs). Blood miRNAs are marked in light orange, and those that were not found are shown in gray. Asterisks indicate genes with miRNAs that were differentially expressed only when lepromatous (LL) was compared with healthy subject. Upregulated miRNAs controlling *IL1A, IL1R1, IGF1R, NOTCH1, JAG1, IL15R, TLR4, CYP27B1, TRAM, NFKB1*, and *IFNB*, and downregulated miRNAs regulating the expression of *CDC42, VAC14, ERBIN, KLF11, PTEN*, and *GRB2*, all in LL patients, demonstrated an immunosuppressive phenotype controlling actin rearrangement and cellular polarity, including phagosome formation, vitamin D antimicrobial activity, cellular differentiation and proliferation, and diverse pathways to stimulate TGF-β-related genes while suppressing NFκB inflammatory functions.

The metalloproteinase ADAM9 and the integrin ITGA5 are two transmembrane proteins involved in mycobacteria invasion of macrophages (21, 22) and endothelial cells (23). Phagocytosis of *M. leprae* may be stimulated by IGF1R (24), LRP1 (25), and PIK3CA (26). In blood, with the exception of *hsa-miR1291*, all miRNAs were downregulated when LPs were compared with HS, indicating that the phagocytosis of *M. leprae* in LP blood was not inhibited. Furthermore, miRNAs that control *IGF1R* gene were upregulated in lesional tissue of LL patients in comparison to HS (Figure 6), which together with the decrease in *IGF1* gene in LL patients (27) may result in inhibition of the microbicidal function of macrophages against *M. leprae* (15) in tissue.

VAC14 is induced to control vacuolation in macrophages (28), while APP produces the oxidative burst (29) and PIK3CA stimulates *M. leprae* phagocytosis (26). Several miRNAs control the expression of *VAC14* and *PIK3CA* genes in lesional tissue and *APP* gene in blood, all of which were downregulated in LP in comparison to HS (Figure 6). When LL patients were compared to HS, only miRNAs that control *VAC14* gene were downregulated, indicating that *VAC14* gene expression was necessary for vacuole formation in LL patients.

Protein kinase C epsilon encoded by the *PRKCE* gene was also found to have downregulated miRNAs in LP. PRKCE is coupled to TLR-4, which is responsible for *M. leprae* recognition (30). Upon phosphorylation, two pathways may be activated: *IRF3* gene upregulation may result in the production of IFN-β and IL-10, especially in LL patients (31), or NFκB upregulation, with the production of proinflammatory cytokines, such as TNF-α and IL-6, by NFκB activation (30). IFN-γ and TNF-α may disrupt the TGF-β pathway by SMAD7 activation followed by TGFBR1 downregulation (32).

Leprosy patients miRNAs control *TGFBR1, SMAD7* gene and the zinc finger transcription factor *KLF11* gene, which regulates *SMAD7* expression. LL patients showed a downregulation of miRNAs that control *KLF11* gene expression, which may result in *SMAD7* gene inhibition and an increase in *TGFBR1* gene, with more TGF-β capture contributing to the immunosuppressive profile of LL. SMAD7 also stimulates CHUK, which inhibits the NFKB1 and COX2 inflammatory pathway. Interestingly, the central inflammatory player, NFKB1, was also found to be regulated by miRNAs in LL patient lesions (Figure 6). It has been previously demonstrated that TGF-β secretion is augmented in LL patients (33) and is secreted by CD4+ CD25+ FOXP3+ T regulatory cells (34), while TGF-β receptors are also increased in lesions of LL patients (35). Additionally, miRNAs that regulated cytoplasmic protein ERBIN were downregulated in LL patient lesions, indicating that ERBIN may also regulate *TGFBR1* gene pathway expression (Figure 6).

**Figure 7:**
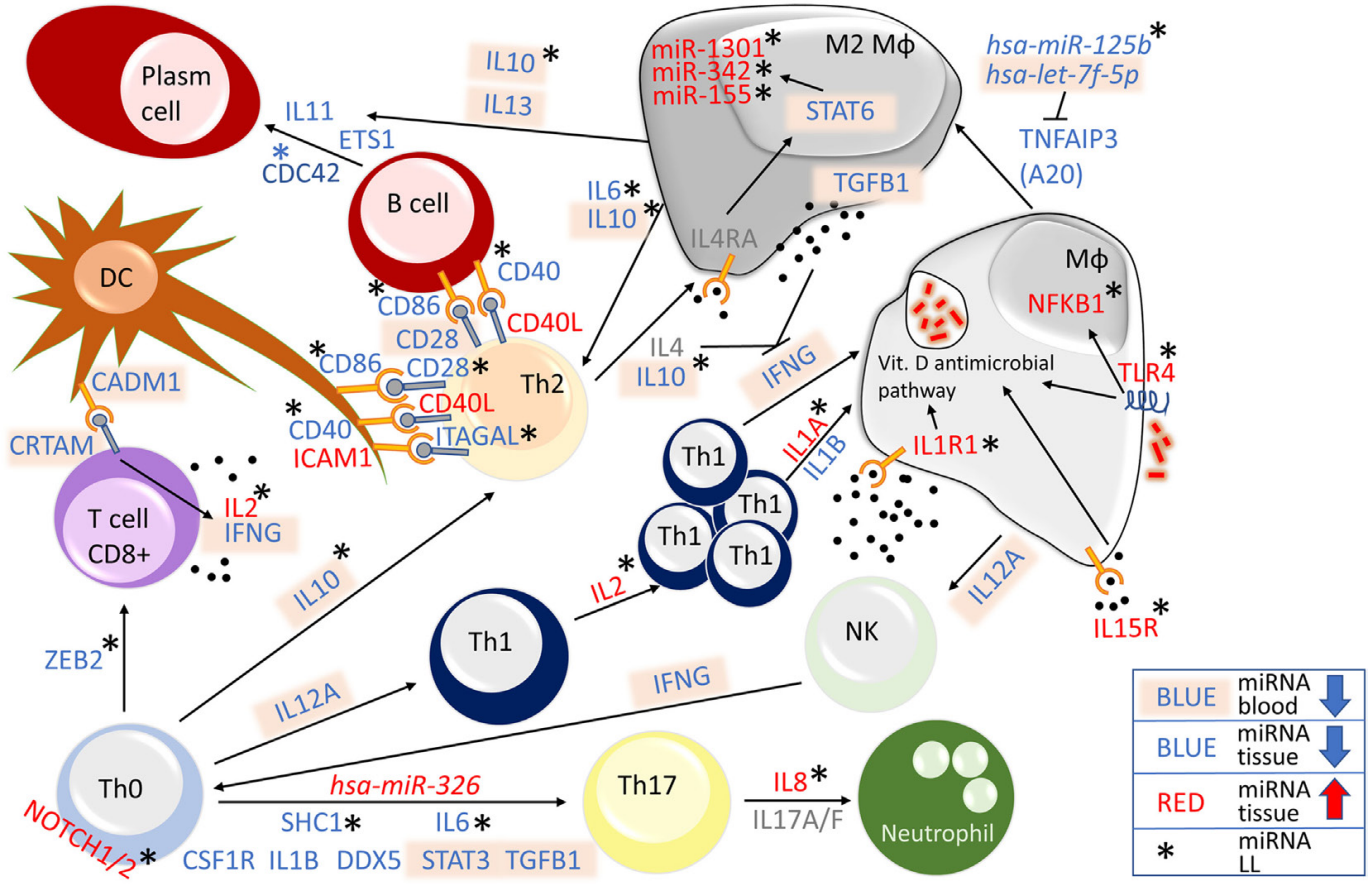
Overview of microRNA (miRNA)-regulated genes related to the immune system in leprosy lesions and blood. Genes regulated by differentially expressed miRNAs were found in blood (all downregulated miRNAs are shown in blue with text marked in light orange) or lesional tissue (red upregulated, blue downregulated). Differentially expressed genes regulating miRNAs (shown in gray) were not found. Lepromatous (LL)—asterisk—upregulated miRNAs for *NOTCH1* and *NOTCH2, IL2, IL8, IL1A, IL1R1, IL15R, TLR4*, and *NFKB1*, and downregulated miRNAs for *ZEB2, SHC1, IL6, IL10, CDC42, ITAGAL, CD40*, and *CD86*, all in LL patients, indicate an immunosuppressive phenotype. Additionally, corroboration of the expression of *hsa-miR-326*, which was upregulated in leprosy patient (LP), *hsa-let-7f-5p*, which was downregulated in LP, *hsa-miR-1301, hsa-miR-342*, and *hsa-miR-155*, all of which were upregulated in LL, and *hsa-miR-125b*, which was downregulated in LL, drove the immune system of LL patients toward an immunosuppressive stage.

NOTCH1/2 have different functions in immune regulation, but overall seem to stimulate the immune system participating in the differentiation of naïve T cells (36) and modulating inflammation (37). An important regulator of M1 macrophage differentiation and the Th1 T cell profile in leprosy, the transmembrane protein NOTCH1 (38) was also found to be regulated by LP miRNAs in lesional tissue of LL patients. Additionally, *NOTCH1 gene, hsa-miR-34a-5p* also control *NOTCH2* and *JAG1* gene in LL lesions (Figures 6 and 7). NOCTH1 is known to be activated by JAG1 on endothelial cells, regulating the differentiation of M1 macrophages (38) in PB leprosy. Both NOTCH1 and NOTCH2 are expressed on Th0 cells and are related to Th17 differentiation (39). Furthermore, NOTCH1/2 are expressed on activated Th1 cells and are critical to the protective response against *Leishmania major* infection by the production of IFN-γ (40), which is also important for leprosy protection by JAG1 stimulation (38).

Activation of TLR4, IL15R, IL1R1, and IL1A is important for antimicrobial activity, a key function for infection control. LL patients were found to have upregulated miRNAs for all those genes in lesional skin (Figures 6 and 7). TLR4 and IL15R converge to CYP27B1, which converts 25-hydroxyvitamin D (25D) to the active hormone 1,25 dihydroxyvitamin D (1,25D) and links to the vitamin D receptor, resulting in the expression of antimicrobial peptides (9, 41). *IL1R1* and *IL1A*, and *TLR2* gene were found to have upregulated miRNAs for all LP, which are also involved in pathways culminating in antimicrobial activity (9). Although *has-mir-21* seems to be critical for the control of the *TLR4, IL15R, IL1R1*, and *IL1A* gene expression in a cell culture-based systems, using our human approach, other miRNAs seemed to be more relevant; however, additional studies are necessary for validation.

TNFAIP3 (A20), which is produced by macrophages infected by *M. tuberculosis*, has been recently described as a new NFκB blocker (42). We found that *hsa-miR-125b* is downregulated only in LL, and *hsa-let-7f-5p* is downregulated in LP (Figure 7). Both miRNAs inhibit *A20* gene expression, leading to an increase in NFκB production. According to our LP miRNA profile, *hsa-miR-125b* and *hsa-let-7f-5p* expression are decreased and therefore do not block *A20* gene, resulting in NFκB abrogation. This phenomenon may drive macrophages toward a M2 profile, with more *TGFB1* (43), *IL6* (44), and *IL10* gene (45) production (all with downregulated miRNAs, Figure 7) that may stimulate Th2 cells to produce more IL-4 (44). Upon ligation, the IL-4 receptor activates STAT6, stimulating the transcription of *miR-1301, miR-342*, and *miR155*, which supports M2 by activating *BCL2* gene and promotes Th2 activation (46). All three miRNAs were found to be upregulated in LL patients, confirming their importance in driving LP toward a Th2 profile.

In addition, *IL10 gene*, with miRNA downregulated only in LL patients, *IL13* gene miRNAs were downregulated in the blood of all LP. Together with lesional *IL11, ETS1* and *CDC42, IL10*, and *IL13* gene induce the differentiation of B cells from plasma cells (47–50). The interaction of CD40 with CD40L results in IL-12 production, which is impaired in LL patients (51). In addition to ICAM1, which has been demonstrated to be inhibited in LL (52), *CD40L gene* miRNAs were upregulated in all LP, and the control of *IL2, IL1A, IL1R, TLR4*, and *NFKB1* gene miRNAs were upregulated in LL (Figure 7), These phenomena may lead to increased *IL4, IL10*, and *TGFB1* production, blocking *IFNG gene* expression (53–56) and resulting in the impaired CIR observed in LL patients.

MicroRNAs targeting the CD8^+^ T cell differentiation gene *ZEB2* gene and activation genes *CADM1* and *CRTAM* gene were found to be downregulated in LP patients (Figure 7). The adhesion molecule CADM1, which is expressed on dendritic cells (DCs), induces a CD8^+^ cytotoxic profile upon ligation to CRTAM T-cells, with the release of IL2 and IFNG (57, 58). Although *IFNG* gene miRNAs were downregulated, *IL2 gene* miRNAs were upregulated in LL patients, suggesting a posttranscriptional blocking of *IL2* released from CD8+ T cells in LP.

Considering Th1, Th2, and Th17, we detected miRNAs controlling all three axes in LP. On the Th1 axis, although *IL12A* and *IFNG* gene miRNAs were downregulated in blood, *IL2 gene*, a key cytokine for Th1 proliferation (59), together with *IL1A, IL1R1, IL15R, TLR4*, and *NFKB gene*, were all found to be regulated by miRNAs in the lesional skin of LL patients. For Th2, *IL6*, and *IL10 gene*, together with the miRNAs *hsa-miR-125b, miR-1301, miR-342*, and *miR-155*, were all regulated by miRNAs, maintaining a suppressive profile in LL patients. Th17 differentiation is influenced by different factors. We found that *SHC1 gene*, which activates *STAT3 gene*, and *IL6* gene that can induce Th17 differentiation in association with *TGFB1gene* (60), both had downregulated miRNAs in LL patients, together with upregulation of the miRNA *hsa-miR-326*, which was described as a Th17 inductor. Although Th17 is known to produce IL8 (61), we found miRNAs controlling the expression of this chemokine in lesions of LL patients (Figure 7), corroborating the absence of this chemokine in polymorphonuclear cells of LL patient unstimulated blood (62).

### Apoptosis

**Figure 8:**
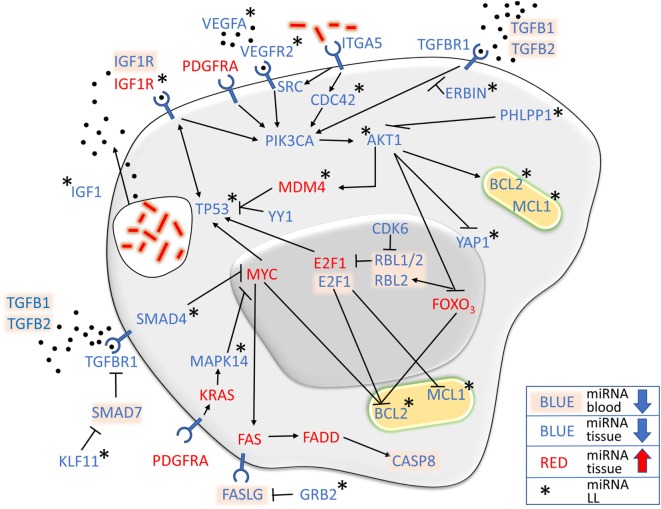
MicroRNA (miRNA) expression strictly controls apoptosis-related pathways. In addition to the antiapoptotic genes *BCL2* and *MCL1*, which were found predominantly in lepromatous (LL) lesions, other genes had downregulated miRNAs in pathways that stimulate their expression, such as *PIK3CA* and *AKT1*, while their suppressors, such as *MYC* and *FOXO3*, had upregulated miRNAs, suggesting an antiapoptotic profile of leprosy patient, especially LL.

The role of apoptosis during *M. leprae* infection is not clear, and different research settings have demonstrated both anti- (24, 63) and proapoptotic (64, 65) features, in addition to possible differences depending on the clinical form of leprosy (66). BCL2 has been shown to be highly expressed in LL patients (66), and *BCL2* and *MCL1 gene* are induced by *M. leprae* on monocytes (63), while CASP8 activity in LL is decreased (67). We found several miRNAs controlling apoptosis pathways in LP. In addition to the downregulation of all miRNAs acting directly on the antiapoptotic gene *BCL2* and its family member *MCL1*, especially in LL lesions, the proapoptotic *gene CASP8* inducers *MYC, FAS*, and *FADD gene* were found to have upregulated miRNAs in LP, while the *FASLG* inhibitor *GRB2* (68) presented downregulated miRNAs in LL patients (Figure 8).

AKT1 and PIK3CA kinases appeared to be central molecules involved in the apoptosis pathway related to *M. leprae*. In contrast, *PIK3CA* miRNAs were downregulated in all forms of leprosy in comparison to HS, and *AKT1* miRNAs was more prominent in LL patient lesions (Figure 8). Interestingly, PHLPP1, a serine-threonine family member that has never described in leprosy and that controls AKT1 (69), was found to have downregulated miRNAs in LL lesions, indicating that *PHLPP1 gene* may have a role in apoptosis control in LP. Furthermore, AKT1 blocks the expression of the proapoptotic *genes YAP1* and *FOX03*, while stimulate the antiapoptotic *gene MDM4*. YAP1 has a proapoptotic function after DNA damage of tumor cells (70), and FOXO3 blocks BCL2 (71). In addition to being inhibited by AKT1, *FOXO3 gene* was found to have upregulated miRNAs in all LP cases (Figure 8). Upon *FOXO3* blockade, *RBL1/2* are transcribed and indirectly block *TP53* expression by blocking *E2F1* (72). MDM4, P53 regulator is also stimulated by AKT1. miRNAs found in our miRNome regulate *FOXO3* and *E2F1* in all forms of leprosy, while miRNAs for *YAP1, MDM4*, and *TP53* were found exclusively in LL. Taken together, these findings show that the influence of LP miRNAs of those pathways in leprosy may result in an antiapoptotic profile.

Considering the miRNA profile controlling cell receptors, in addition to *FAS gene*, we found five other genes related to apoptosis control by miRNA in *M. leprae* infection: *TGFBR1*, involved in TGF-β signaling, a cytokine known to induce tolerance (73, 74) with a suppressive potential of Tregs in LL patients (75); *ITGA5*, an α-integrin linked to *M. tuberculosis* infection of macrophages (22); *VEGFR2*, known to participate in *M. tuberculosis* dissemination by triggering angiogenesis (76), while its ligand, *VEGFA*, has been demonstrated to be expressed in leprosy lesions (77); *PDGFRA*, which was shown to be upregulated in SC 27 days after *M. leprae* infection (78), and its ligand *PDGF*, a potential marker for erythema nodosum leprosum (79); and *IGF1R*, the receptor of *IGF1*, which inhibits macrophage and SC apoptosis upon *M. leprae* infection, in turn stimulating the production and secretion of *IGF1* (15).

In contrast to downregulated miRNAs controlling *PIK3CA, AKT1, BCL2*, and *MCL1*, miRNAs controlling the expression of the proapoptotic genes *MYC, E2F1*, and *FOXO3* were all found to be upregulated in lesional tissue. Additionally, miRNAs for *FAS* and *FADD*, members of the *CASP8* proapoptotic pathway, were also found to be upregulated (Figure 8). Taken together, concerning miRNA regulation, our data suggest an antiapoptotic profile for leprosy in general, driven by *BCL2, MCL1*, and *CASP8*.

### SCs, Demyelination, and EMT

Demyelination is a pathologic process that destroys the myelin sheath and involves multiple factors, including inflammatory responses or infections (80). LP demyelination is the ultimate consequence of leprosy neuritis, and LL patients exhibit myelinated and non-myelinated SC infected by *M. leprae* (81).

**Figure 9:**
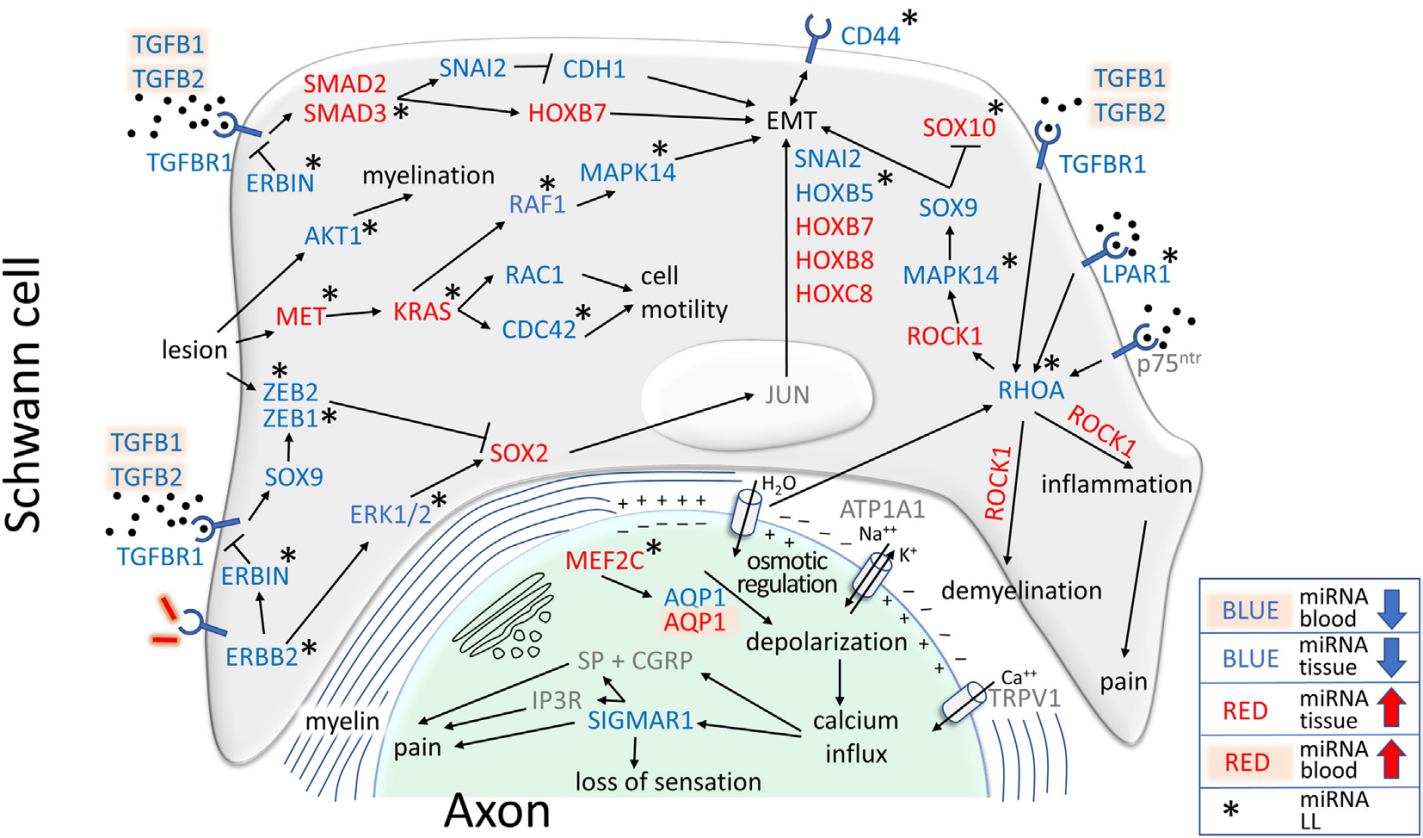
MicroRNAs (miRNAs) related to the epigenetic control of genes involved in demyelination, epithelial–mesenchymal transition (EMT), inflammation, pain, and loss of sensation were differentially expressed in leprosy patient (LP). Different pathways, including canonical and non-canonical pathways of TGF-β, may drive cell toward EMT, with a Schwann cell (SC) undifferentiated phenotype consisting of the absence of p75^ntr^, in gray, *SOX10*, with upregulated miRNAs in lepromatous (LL) and blockade by the *SOX9* pathway, and the presence of *CD44*, which had downregulated miRNAs in LL. *RHOA* miRNAs were downregulated in LL, stimulating *ROCK1* to drive EMT, inflammation and pain, which was controlled by upregulated miRNAs in all LP. *AQP1*, the only gene with upregulated miRNAs in blood in LP and downregulation in lesional skin, is one of the genes responsible for osmotic regulation. In lesions, downregulated miRNA for *AQP1* indicated that *AQP1* might not be expressed in the nerve, which was consistent with the upregulation of miRNAs for the *MEF2C AQP1* transcription factor, possibly resulting in depolarization and calcium influx stimulating *SIGMAR1*, which also had downregulated miRNAs. The low expression level of *SIGMAR1* may result in loss of sensation, but its overexpression may result in pain. Depolarization may stimulate *RHOA* to maintain a cycle of demyelination, inflammation, loss of sensation, and pain.

Upon invasion, *M. leprae* stimulates *ERBB2* independently of *ERBB3* (82), resulting in *ERK1/2* activation, which leads to peripheral nerves demyelination (83). *ERBB2* miRNAs were downregulated in LL patients (Figure 9), indicating a possible role for the *SOX2* and *JUN* pathway in demyelination and EMT (78). However, *SOX2* miRNAs were found to be upregulated in all LP, while *ZEB1/2* miRNAs were downregulated in LL patients (Figure 9), indicating that ZEB1/2 may regulate SOX2 (84) expression to inhibit demyelination and EMT. Although *ZEB1* expression increases after *M. leprae* infection or after TGF-β stimulation (85), while ZEB2 is essential after nerve injury by allowing remyelination and functional recovery (86), they may be regulated by ERBIN, which blocks the TGFBR1 pathway (87), and the downregulated miRNAs were observed only in LL patients (Figure 9).

Schwann cell lesions induce the antiapoptotic molecule *AKT1* and the mitogen *MET*, both of which are regulated by miRNAs in LL patients. AKT1 signaling, one of the most important pathways involved in myelination (88), may be activated in injured peripheral nerves (89). We found that miRNAs for *AKT1* were downregulated in LL patients, indicating a possible role for *AKT1* in LP myelination (Figure 9). In parallel, MET, an important inducer of neural injury (90) and the *KRAS, RAF1*, and *MAPK14 genes* involved in EMT pathways (91) were found to have miRNAs in LL patient lesions. In contrast, *MAPK14* and *RAF1 genes* had downregulated miRNAs, and *MET* and *KRAS genes* had miRNAs upregulated, indicating a strict control of the first steps of the pathway. *MET* and *KRAS* also regulate cell motility through RAC1 and CDC42, which are important for actin rearrangement and cellular polarity (92).

Very few works have described the effects of *HOX* and *SNAI genes* on leprosy pathophysiology. It has been demonstrated that upon *M. leprae* infection, SC may switch off differentiation genes, such as *SOX10* and *p75^ntr^*, while switching on EMT genes, especially the *HOX* family (78) and *CD44*, which are considered a marker for EMT in SC (78), with downregulated miRNAs in our LP skin lesion samples. Our study did not find miRNAs regulating *p75^ntr^*, which is compatible with the switching off demonstrated in the previous work; however, we detected upregulated miRNAs for *SOX10* only in LL, downregulated miRNAs for *SNAI2* and *HOXB5*, with the latter only in LL patient lesions, and upregulated miRNAs for *HOXB7, HOXB8*, and *HOXC8* in all LP (Figure 9). Interestingly, the only genes that were detected in the earlier *in vitro* work and that for which we also detected miRNAs were *SNAI2* and *HOXB8*. miRNAs for *HOXB5, HOXB7*, and *HOXC8* were found in our work, but their expression was not detected previously.

*HOXB5 gene* is a marker for long-term hematopoietic stem cells (93) and it affects the differentiation of the vascular endothelium development from precursor cells (94). It is known that endothelial cells are important for the entry and maintenance of *M. leprae* in nerves (95) and that vasculitis may be observed in leprosy reactions, with endothelial proliferation in Lucio’s phenomena (1). *HOXB7* is associated with EMT in breast cancer cells *via* the canonical TGF-β pathway (96), while *HOXC8* mutant mice present motoneuron abnormalities with analog molecular defects compared with mutant mice for retinaldehyde dehydrogenase 2 synthesizing enzyme (97), which is responsible for retinoic acid synthesis, with atrophy of the distal projections of the ramus profundus of the radialis nerve that supply the extensor muscles of the forepaw, resulting in forepaw neuromuscular defects. Vitamin A levels in LP have been shown to be low in comparison to healthy controls, and much lower in LL patients (98).

*SNAI2* and *HOXB8* have been demonstrated to be upregulated in SC infected with *M. leprae in vitro* (78). SNAI2 is activated by the canonical TGF-β pathway (99), inhibiting CDH1 (E-cadherin) and resulting in cellular EMT (100). We found that upregulated miRNAs controlled *SMAD2* and *SMAD3* expression in LP. *SMAD3* miRNAs were significantly upregulated only in LL patients, possibly participating in the control of SC-EMT (Figure 9). *HOXB8* null mutants show altered sensory responses in mice, probably due to a smaller number of neurons and neural disorganization (101), indicating that *HOXB8* expression levels may be related to sensory alterations in LP.

### Loss of Sensation and Neuropathic Pain in Leprosy

ROCK1 is a Rho-associated protein kinase that is present in different signaling pathways in neurons (102), is known to regulate SC myelination (103), and may be activated by *RHOA gene* (104), for which we found downregulated miRNAs in LL lesions. *ROCK1 gene* stimulated by TGFBR1 induced EMT of SC *via* the MAPK14 pathway, which leads to SOX9 activation and SC EMT associated with the blockade of SOX10 (Figure 9), a recognized inducer of cell differentiation (105). We observed upregulated miRNAs for *SOX10 gene* in LL lesions, indicating that it may be expressed in LL patients. Myelin-associated inhibitors upon ligation with p75^ntr^ activate *RHOA*, resulting in demyelination through *ROCK1 genes* (106), while *ROCK1* stimulated by *BDNF* through p75^ntr^ and *RHOA* may lead to inflammation and pain (107).

LPAR1 signaling is required to initiate neuropathic pain after nerve injury. Mice lacking *LPAR1 gene* do not present signs of neuropathic pain, and inhibition of *RHOA* and *ROCK1* also prevent neuropathic pain (108). We found that miRNAs for *LPAR1* were downregulated in LL patients in both blood and lesions, indicating that the receptor may be available for ligation in LP. *RHOA* miRNAs were also downregulated in LL patients, while *ROCK1* was upregulated in all LP, indicating an attempt to control EMT, demyelination and pain.

The miRNA *hsa-miR-1291* was the only differentially expressed miRNA in both skin tissue and blood samples. It was predicted to regulate the aquaporin-1 (*AQP1*) gene (109), which influences the hydration, elasticity and glycerol permeability of skin (110). In LL lesions, frequent overexpression of lipid metabolism genes (111) indicates that *M. leprae* uses host lipids for growth and virulence. Therefore, downregulation of *hsa-miR-1291* in skin lesions could modulate *AQP1* expression and increase glycerol permeability to promote fatty acid metabolism. Altered *AQP1* expression may improve our comprehension of some well-known clinical issues related to leprosy, such as the dryness found in skin lesions. Moreover, *MEF2C*, a transcription factor for *AQP1* (112), was found to have upregulated miRNAs in the skin lesions of LL patients, which could result in an absence of *AQP1* transcription in LL patients, contributing to loss of sensation.

Aquaporins may be key molecules in leprosy pathophysiology. *AQP1* knockout mice have impaired pain sensation (113), and human trigeminal neurons that mediate head nociception and innervate the oral mucosa express *AQP1*, indicating an involvement in sensory transduction (114). Peripheral nerve system expression of *AQP1* has been seldom investigated, but it has been shown in the sciatic nerve (115) and in Ruffini mechanoreceptors (116). *AQP4* is expressed in the olfactory epithelium (117) and in retinal glia (118), and it is the target of anti-AQP4 antibodies in autoimmune neuromyelitis optica (119). Loss of sensation is the hallmark of leprosy, but there is no definite mechanism explaining this phenomenon.

*AQP1* participates in the mechanism of thermic and chemical pain, likely controlling neuronal ionic nociceptive homeostasis (113). Membrane depolarization activates *ATP1A1*, which regulates sodium potassium channels, and *TRPV1*, which is responsible for calcium influx into the cell. Calcium acts on calcitonin gene-related peptide (CGRP), which together with substance P (SP) results in pain (113). In addition to CGRP, calcium also stimulates *SIGMAR1* (120), for which we detected downregulated miRNAs in LP lesional skin (Figure 9). *SIGMAR1* acts as a chaperone for *IP3R* to maintain calcium signaling from the endoplasmic reticulum to mitochondria, and it has been implicated in pain (121). In contrast, *SIGMAR1* agonists potentiate pain, antagonists potentiate analgesia (122), and neuropathic pain was strongly attenuated in *SIGMAR1* knockout mice (123). Interestingly, *AQP1* silencing in tumor cells abrogates the expression of *RHOA* and *TGFB1/2* (124), indicating a possible mechanism to maintain EMT, demyelination, inflammation and pain through *AQP1, SIGMAR1, RHOA*, and *ROCK1* in leprosy.

Taken together, our data suggest an important role for miRNA expression in leprosy immunophysiopathology, especially the regulation of different parameters of the immune system, apoptosis, SC demyelination, EMT, and neuropathic pain. The epigenetic control of the genes expressed in leprosy lesions and blood by miRNAs may provide new insights into the different facets of leprosy, from *M. leprae*–host cell interactions to new therapeutic targets.

## Ethics Statement

This study was carried out in accordance with the recommendations of Brazilian National Ethics Committee (CONEP) guidelines, approved by Pará Federal University Ethics Committee number CAAE 26765414.0.0000.0018, with written informed consent from all subjects. All subjects gave written informed consent in accordance with the Declaration of Helsinki. The protocol was approved by the Pará Federal University Ethics Committee.

## Author Contributions

CS, PP, JB, MS, SS, and AR designed research; CS, RB, AG, AM, and MF enrolled patients, performed, and registered clinical diagnosis; CS, PP, TS, AS, FM, AV, SS, and AS performed research; CS, PP, RB, AG, AM, TS, AS, FM, AV, LG, JS, SS, and AS analyzed the data; CS, PP, RB, AG, AM, JS, SS, and AS wrote the article; CS, PP, RB, AG, AM, TS, AS, FM, AV, LG, JB, MS, MF, JS, SS, and AS agree with manuscript results and conclusions.

## Conflict of Interest Statement

The authors declare that the research was conducted in the absence of any commercial or financial relationships that could be construed as a potential conflict of interest.
